# Behavioral, Physiological, Demographic and Ecological Impacts of Hematophagous and Endoparasitic Insects on an Arctic Ungulate

**DOI:** 10.3390/toxins12050334

**Published:** 2020-05-20

**Authors:** Kyle Joly, Ophélie Couriot, Matthew D. Cameron, Eliezer Gurarie

**Affiliations:** 1 Gates of the Arctic National Park and Preserve, Arctic Inventory and Monitoring Network, National Park Service, Fairbanks, AK 99709, USA; matthew_cameron@nps.gov; 2 National Socio-Environmental Synthesis Center, SESYNC, 1 Park Place, Suite 300, Annapolis, MD 21401, USA; ocouriot@sesync.org; 3 Department of Biology, University of Maryland, College Park, MD 20742, USA; egurarie@umd.edu

**Keywords:** *Aedes* spp., behavior, caribou, demographics, energetics, *Hypoderma tarandi*, mosquito, movement, physiological, warble fly

## Abstract

Animals that deliver a toxic secretion through a wound or to the body surface without a wound are considered venomous and toxungenous, respectively. Hematophagous insects, such as mosquitoes (*Aedes* spp.), meet the criteria for venomous, and some endoparasitic insects, such as warble flies (*Hypoderma tarandi*), satisfy the definition for toxungenous. The impacts of these insects on their hosts are wide ranging. In the Arctic, their primary host is the most abundant ungulate, the caribou (*Rangifer tarandus*). The most conspicuous impacts of these insects on caribou are behavioral. Caribou increase their movements during peak insect harassment, evading and running away from these parasites. These behavioral responses scale up to physiological effects as caribou move to less productive habitats to reduce harassment which increases energetic costs due to locomotion, reduces nutrient intake due to less time spent foraging, and can lead to poorer physiological condition. Reduced physiological condition can lead to lower reproductive output and even higher mortality rates, with the potential to ultimately affect caribou demographics. Caribou affect all trophic levels in the Arctic and the processes that connect them, thus altering caribou demographics could impact the ecology of the region. Broadening the definitions of venomous and toxungenous animals to include hematophagous and endoparasitic insects should not only generate productive collaborations among toxinologists and parasitologists, but will also lead to a deeper understanding of the ecology of toxic secretions and their widespread influence.

*“The buck, who had been standing motionless at the opposite end of the gravel bar all this time, suddenly exploded into action, showing all the typical reactions to an attack by a nose or warble fly. He threw up his head, stamped his feet, twitched his hide, and then began to gallop along the beach, bucking and tossing as he ran. He suddenly stopped and stood stock still. Then he wheeled, galloped back in his tracks, stopped again, shook his head, wheeled once more and galloped full speed toward the band at the water’s edge. He literally burrowed his way into their midst where l lost him and presumably the nose fly did likewise.”*

—W. O. Pruitt [1]

## 1. Introduction

Animals that produce a secretion used on another animal that disrupts physiological or biochemical processes in order to facilitate behaviors such as feeding or defense can be considered venomous if delivered through a wound, or toxungenous if delivered to the body surface absent a wound [2,3]. While sometimes overlooked in this regard, hematophagous insects such as the mosquito (*Aedes* spp.) and endoparasitic insects such as the warble fly (*Hypoderma tarandi*) have the requisite characteristics to be broadly characterized as venomous and toxungenous, respectively. Mosquitoes, like all hematophagous animals, secrete fluids to help them obtain blood [4,5]. After the mosquito’s proboscis enters its target, it begins secreting saliva that has platelet aggregation inhibitors, like the enzyme apyrase, to prevent platelets from coming together to form blood clots as well as anticoagulant properties, which slow the formation of clots, help induce hematomas, and improve the chances of acquiring a blood meal [6,7]. Warble larvae secrete protease and other enzymes to create a wound in order to enter its host skin, which is an example of toxungen rather than a venom [3]. However, the larvae secrete enzymes while traveling through its host’s body and the enzyme hypodermin C, which is used for the hydrolysis of the host’s proteins that are then used as nutrients for the larvae while it is inside its host [8,9].

Arctic mosquitoes are in diapause over the winter. Snow and ice melt trigger the hatching of mosquito eggs, after which the larvae feed on vegetation [10]. From emergence (late May to mid-June) to cessation of activity (late July), adult females seek blood meals, which will allow them to reproduce. During this time period, mosquito numbers are remarkable, with an estimated 17 trillion individuals in Alaska alone [11,12]. Mosquito abundance is positively related to warmer summer temperatures, while their activity level is tied to low wind velocities because they are relatively poor fliers [10,13,14].

Warble flies are much less numerous than mosquitoes. However, almost every caribou and reindeer (both *Rangifer tarandus*; henceforth referred to as just caribou) is afflicted by them and exhibit stronger responses to them than from mosquitoes [10,15,16,17]. The presence of even a single warble fly can elicit strong responses by caribou [1,11]. While warble fly abundance and activity levels are also positively associated with warmer summer temperatures, they are strong fliers and thus less dependent on calm conditions [14,18]. Unlike mosquitoes, adult warble flies do not directly seek nutrients from its host. Rather, adults lay their eggs on the hair of caribou in July and August [19,20]. After 3–7 days, the larvae hatch, burrow into the skin of the caribou, migrate in its body and, eventually, the third instar forms nodules and overwinters in its host [8,9]. The mature larvae exit the host in May to June and then mature some weeks later to emerge as adult flies [21].

## 2. Impacts of Mosquitoes and Warble Flies on Caribou

It is hard to overstate the ecological and socio-economic importance of caribou for the Arctic. Caribou span the northern hemisphere and are the most numerous ungulate in the Arctic [22]. Herds of caribou can number into the hundreds of thousands of individuals [23]. Caribou are vital to the culture and economy of the Arctic [24]. Subsistence harvest of caribou remains high in northern regions, with caribou being the most utilized terrestrial resource in many regions. While Westerners have known of the impacts of insects on caribou for more than a century [25], aboriginal peoples must have known for millennia. The impacts of mosquitoes and warbles are obvious, wide ranging, and can be dramatic. The most immediate responses of caribou responses to mosquitoes and warble flies are behavioral, with direct and indirect physiological effects and, ultimately, potential demographic and ecological effects.

### 2.1. Behavioral Impacts

**Figure 1:**
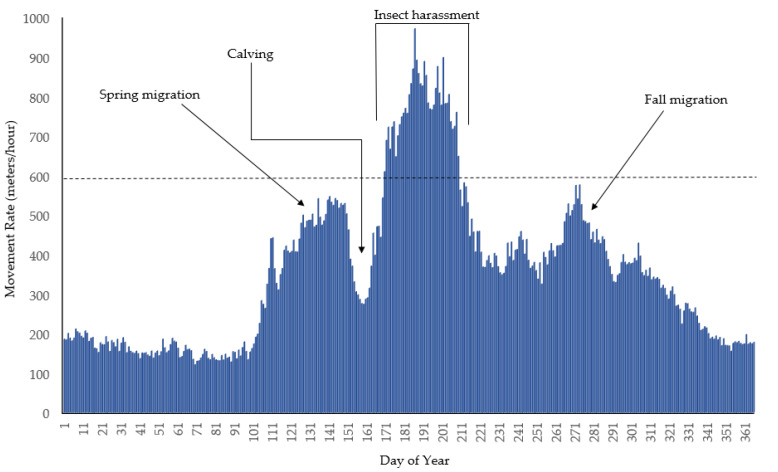
Average movement rates (meters/hour) of Western Arctic Herd female caribou by day of the year, northwest Alaska, 2009–2019.

Behavioral responses to increasing insect harassment are numerous and wide ranging. First, insect harassment is thought to increase caribou movements [26,27,28,29]. For example, average movement rates of Western Arctic Herd caribou, found in northwest Alaska, range from 121 to 971 meters/hours (m/h) over the course of the year (Figure 1). Movement rates are lowest in winter (i.e., beginning of December through the beginning of April) but are elevated for both spring and fall migration. After spring migration, movement rates drop as female caribou give birth and the calves ready themselves to travel with the rest of the herd. Movement rates were consistently the greatest from June 19th to July 28th (also see [30,31,32]), when insect harassment was greatest. This was the only time of the year that movement rates exceeded 600 m/h (Figure 1). This pattern is striking and unexpected, as one might expect that movement rates would be highest during the iconic spring and fall migration periods but are actually greatest in the summer when caribou could be foraging in highly productive habitat to accumulate fat and protein to replenish stores depleted during the winter and aid in nursing their calves.

The increased movement may be a direct response to evade insects [27]. Indeed, caribou are known to travel long distances in order to avoid warble flies [1,11]. This may include diverse strategies such as walking long distances into the wind or evasive actions such erratic movements or swimming. However, this additional time spent moving has a cost, since it leaves less time for feeding, resting and ruminating [33].

**Figure 2:**
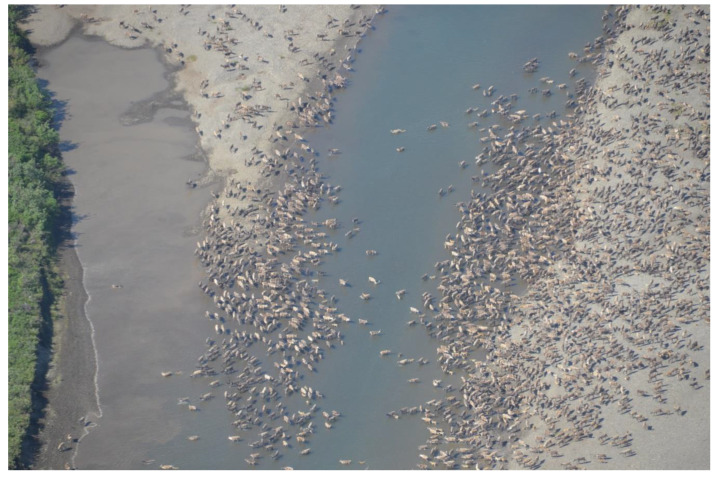
Caribou aggregating on gravel bars and in the river itself in an attempt to reduce insect harassment, northwest Alaska. Time spent in insect relief habitat reduces the amount of time spent foraging. Photo: K. Joly (NPS).

Beside the tradeoff between insect avoidance and time spent on other essential activities, insect harassment can also impact the quality and the quantity of forage accessed by caribou. Often these insect-related movements will drive caribou to use insect relief habitats (Figure 2), such as windswept or snow-covered areas, or even gravel bars, lakes or ocean shallows [11,13,27,28,34,35,36,37]. Typically, these insect relief habitats have very low vegetative productivity. Moreover, the abundance of these habitats can be limiting. The result is that caribou can form extremely large (>100,000 individuals) and tight aggregations in these habitats during peak insect harassment [38]. This increases competition for resources and diminishes the quantity of forage intake per capita. In addition, these large aggregations of caribou can have a direct impact on the parasitic load of some individuals, since social status can influence the spatial location of individuals in these aggregations and thus their relative susceptibility to infestation [39]. Caribou found in the interior of an aggregation are exposed to relatively less insect harassment, as they are surrounded by and often physically touching other caribou, than caribou along its perimeter or off by themselves. Finally, as insect harassment impacts caribou habitat selection, avoiding areas with high parasites can also alter use of foraging areas and the distribution of caribou across the landscape [40]. It has also been hypothesized that caribou will move away from calving areas, where warbles drop out of caribou and hatch, in order to reduce harassment from the adult flies [39,41]. The impacts of these movements are not fully understood.

### 2.2. Physiological Impacts

All reported insect harassment-driven behaviors that caribou display can have direct or indirect physiological impacts. The greater movements increase energetic expenditures [42] and the reduced feeding time, in addition to lower forage quality and quantity due to altered habitat selection and large aggregations during the short arctic growing season, directly act to reduce body condition and/or growth rate. This is especially true for females that have the added demand of lactation, individuals already in poor condition, and on rapidly-developing calves [13,40,43]. Following summers with conditions that allow for high levels of insect harassment, females tend to reach their calving grounds later [44]. The indirect impacts of these late arrivals on calf survival and replenishing protein stores of parturient females represents a knowledge gap and a promising area of future research.

Male caribou tend to have greater warble larvae loads than females [15,17,45]. Poor growth during the first year of life of male calves can have long-term indirect impacts for entire cohorts born in a high insect harassment year, as body size helps determine competitive abilities during the rut [43]. However, some caribou calves have been known to exhibit compensatory growth during the winter [46].

Heavy warble infestations can also directly reduce weight gain [15,29,47,48] and limit the amount of stored fat caribou carry into the winter season [16,40]. These extra burdens add to the other direct and indirect costs associated with increased parasitic insect loads. Direct physiological impacts of the parasites include costs associated with biochemical and inflammatory immune responses by the caribou and the siphoning off of nutrients and/or blood (blood loss has been estimated to reach up to 125 g/day), that can also lead to a lower body condition [8,9,18,40,49]. Host responses are designed to reduce the impacts of the parasites or even, in the case mosquitoes, kill them [6,7]. Thus, parasites have co-evolved by developing strategies to lower the amount of time required to breach their host [6,7,50].

### 2.3. Demographic Impacts

The behavioral and physiological responses of caribou to insect harassment can have demographic and population-level consequences. Females with lower body conditions are less likely to become pregnant [51,52] and heavy warble fly loads have been associated with lower pregnancy rates in caribou [40]. Therefore, particularly bad years of insect harassment can impact entire cohorts [43]. Moreover, extreme warble fly infestations can even lead to lower overwinter survival in calves and delay the timing of calving [16,43,44]. High pregnancy and recruitment rates are vital for population growth. Thus, physiological impacts can have a multiplier effects [53] that lead to demographic impacts in caribou [11,15,16,47,48,54]. In other words, small, sub-clinical changes in the amount of time spent foraging, energy expended for additional locomotion, and intake of high-quality forage accumulated over the course of the short Arctic growing season can make the difference in females becoming pregnant or not and calves surviving over the winter or not [16,55]. All these insect-related impacts would negatively influence caribou populations. Many caribou populations around the world are in decline and these negative influences of insects on caribou demographics are exacerbated during population declines [11].

### 2.4. Ecological Impacts

Caribou herds, which can number into the several hundreds of thousands of individuals, are an ecological force in the Arctic [23]. These herds can dramatically effect vegetation through grazing and/or trampling, reducing abundance of shrubs and lichens [56,57,58]. Caribou exhibit the longest terrestrial migrations on the planet, thus they transport nutrients over huge (>100,000 km^2^) areas which can affect soil nutrient concentrations [57,59,60]. These nutrients come from digestive waste products of caribou but also from their carcasses [59]. Caribou are the primary prey for wolves (*Canis lupus*) but are also sought after by brown bears (*Ursus arctos*), wolverine (*Gulo gulo*), golden eagles (*Aquila chrysaetos*), and other predators [61,62]. Remains of these carcasses are utilized by scavengers, like ravens (*Corvus corax*), and then eventually by decomposers. Thus, caribou affect all trophic levels in the Arctic and the ecological processes that connect them. Since hematophagous and endoparasitic insects have the capability to affect caribou demographics, as well as caribou habitat selection and density in insect relief habitats, they also have the potential to indirectly impact the ecology of the entire arctic ecosystem. Outside the sphere of caribou, mosquitoes have numerous direct impacts on arctic ecology including acting as decomposers (in their larvae stage), pollinators, and food sources for a variety of bird species [63].

## 3. Conclusions

By utilizing broad definitions of venomous and toxungenous that encompass hematophagous and endoparasitic insects, like mosquitoes and warble flies, biologists increase their ability to more fully understand the ecology of toxic secretions and their wide-ranging influence. Similar to other studies, we found that movement rates were greatest during the insect harassment period. We reviewed some, but not all, of the various impacts these insects can have on caribou—the dominant herbivore in northern climes. We suggest a causal mechanism, through behavioral changes (increased movements that lead to greater energy expenditures, reduced foraging time, and increased use of low productivity habitats), that link insect harassment to poorer physiological conditions in caribou which potentially culminate in detrimental demographic consequences. Changes in caribou population sizes can have profound ecological consequences in the Arctic. While pronounced in the Arctic, hematophagous and endoparasitic insects can impact important ungulate species in other biomes [64] as well as arctic mammals other than caribou [38]. Indeed, these types of insects are widespread and impact a wide array of species across the globe including humans, birds, amphibians, and reptiles [65,66,67].

Temperatures have been rising twice as fast in the Arctic than other parts of the world [68]. These increased temperatures are predicted to lead to a numerical increase in hematophagous and endoparasitic insects [10,11]. Thus, warming associated with climate change may further increase the impacts of these insects in the future [11,47]. Warming may also allow the range expansion of other venomous parasites, like the winter tick (*Dermacentor albipictus*), into new areas that potentially could be highly damaging to native fauna [69,70]. The effects of hematophagous and endoparasitic insects may also interact with other disruptions like anthropogenic disturbances, other parasitic insects and predation pressure [11,13], potentially compounding the complexity and degree of impact. While extensive research has been conducted into the influence of hematophagous and endoparasitic insects on ungulates, there are still important knowledge gaps. One promising line of research is scaling up metrics of insect harassment, such as Witter et al. [71], to large geographic areas using remote sensing data and relating these to ungulate ecology. We encourage researchers to think broadly about venomous and toxungenous animals and their far-ranging consequences.

## 4. Materials and Methods

We instrumented over 250 female caribou from the Western Arctic Herd with GPS collars that were programmed to continuously collect relocations every 8 hours from 2009 to 2019 [72]. Animal handling protocols were approved by a State of Alaska Intuitional Animal Care and Use Committee (IACUC 0040-2017-40, approval date: 10 August 2017). Movement rates (meters/hour) of caribou were determined by dividing the distance between successive GPS locations by the duration between them. To determine daily movement rates for each day of the year, movement rates for that day were averaged and the duration between relocations had to be between 7 and 9 hours as the amount of time between relocations affects the estimation of movement rates [73].
